# Volatile Compounds of Raspberry Fruit: From Analytical Methods to Biological Role and Sensory Impact

**DOI:** 10.3390/molecules20022445

**Published:** 2015-01-30

**Authors:** Eugenio Aprea, Franco Biasioli, Flavia Gasperi

**Affiliations:** Department of Food Quality and Nutrition, Research and Innovation Centre, Fondazione Edmund Mach, 38010 San Michele all’Adige (TN), Italy; E-Mails: franco.biasioli@fmach.it (F.B.); flavia.gasperi@fmach.it (F.G.)

**Keywords:** raspberry (*Rubus idaeus* L.), volatile organic compounds, flavor, aroma, glycosidically-bound volatiles, enantiomers, headspace analysis, odor active compounds, *Botrytis*

## Abstract

Volatile compounds play a key role in the formation of the well-recognized and widely appreciated raspberry aroma. Studies on the isolation and identification of volatile compounds in raspberry fruit (*Rubus idaeus* L.) are reviewed with a focus on aroma-related compounds. A table is drawn up containing a comprehensive list of the volatile compounds identified so far in raspberry along with main references and quantitative data where available. Two additional tables report the glycosidic bond and enantiomeric distributions of the volatile compounds investigated up to now in raspberry fruit. Studies on the development and evolution of volatile compounds during fruit formation, ripening and senescence, and genetic and environmental influences are also reviewed. Recent investigations showing the potential role of raspberry volatile compounds in cultivar differentiation and fruit resistance to mold disease are reported as well. Finally a summary of research done so far and our vision for future research lines are reported.

## 1. Introduction

Raspberry (*Rubus idaeus* L.) is a member of the Rosaceae family producing a red fruit with a sweet but tart flavor. Some cultivars with recessive genes giving an extremely low concentrations of anthocyanin produce yellow berries [1], but it is the European red fruited cultivars that are most widely grown and economically most important. Although it is called a berry, the fruit produced by the raspberry is, in botanical terminology, a collection of numerous drupelets around a central core. The drupelets typically separate from the core when pickled.

This commodity is of continuously increasing economic importance, as witnessed by nearly 50 raspberry breeding programs around the world [2] and the ongoing raspberry sequencing project [3].

Red raspberries contain high amounts of polyphenols and antioxidants, and have a unique phytochemical profile rich in ellagitannins and anthocyanins that distinguishes them from other berries and fruits [4] and has positive implications for human health and the prevention of chronic diseases [5], although these fruits are mostly recognized and appreciated for their characteristic flavor. Volatile compounds play a key role in the formation of the flavor of food products and nearly 300 volatile compounds have so far been reported in raspberry. Volatile organic compounds (VOCs) are organic molecules with appreciable vapor pressure at ordinary room temperature. They are usually small molecules with a molecular weight lower than 300 Dalton. People often associate scents with volatiles that can be perceived by the human nose and have a pleasant smell [6] and flavor. It should be mentioned that volatile compounds in plants have various ecological and productive impacts: they attract pollinating insects, advertise that fruit are ripe and ready for seed dispersal, modulate systemic acquired resistance to pests and diseases, and also seem to alleviate abiotic stress [7].

Despite the economic and nutraceutical importance of raspberry, there has been little mention in the literature over last ten years of the volatile compounds in this fruit. A series of studies carried out by Firmenich in the ’60s and ’70s defined the basic methodologies and listed the compounds isolated and identified in raspberries. Of these, 4-(4-hydroxyphenyl)butan-2-one was recognized as the key compound in defining typical raspberry flavor and was therefore named “raspberry ketone”. In the following decades only a few investigations on volatile compounds in raspberry were carried out and very little is known about the real impact the different volatile compounds have on human sensory perception or about their role in pest defense.

This paper reviews the studies carried out on the isolation and identification of volatile compounds in *Rubus idaeus* L. focusing on aroma-related compounds, from the pioneering application of separation methods to the most recent investigations. Table 1 lists all the volatile compounds identified in raspberry up to now, with main references and, where available, quantitative data. Two additional tables report the glycosidic bond and enantiomeric distributions of volatile compounds investigated in raspberry fruit. Studies on volatile compound development and evolution during fruit formation, ripening and senescence, and genetic and environmental influences are also reviewed. Finally, we look at recent investigations showing the potential role of raspberry volatile compounds in cultivar differentiation and fruit resistance to mold disease.

**Table 1:** Reported volatile compounds in raspberry fruit (*Rubus idaeus* L.)

Compounds		Quantitative Data	Refs.	
		(mg/Kg)	Identification	Quantitation *
Acids				
1	2-Hexenoic acid	X	[8,9]	
2	2-Methylbutanoic acid	tr	[**10**]	[**11**]
3	3-Hexenoic acid	X	[8,9]	
4	3-Methylbutanoic acid (isopentanoic acid)	0.05	[8,9]	[**12**]
5	3-Methyl-2-butenoic acid	tr		[**11**]
6	3-Methyl-3-butenoic acid	tr		[**11**]
7	Acetic acid	1.35; 16; 5.4–135; 0.0205–0.275	[8,**10**,**13**,**14**]	[**11,12,15,16**]
8	Benzoic acid	<0.025; tr	[9]	[**11,12**]
9	Butanoic acid	0.25 ; 0.6	[8,9,**10**,**15**]	[**11,12**]
10	3-Phenylprop-2-enoic acid (cinnamic acid)	X	[9]	
11	Decanoic acid	<0.025; tr	[9]	[**11,12**]
12	Dodecanoic acid	X	[9]	
13	Ethylhexanoic acid	X	[9]	
14	Formic acid	X	[8]	
15	Heptanoic acid	0.15	[9]	[**12**]
16	Hexadecanoic acid	Tr		[**11**]
17	Hexanedioic acid (adipic acid)	X	[9]	
18	Hexanoic acid	1.7; 6.7; 0.4–19.3; 0.0286–0.1586	[8,9,**13**,**14**]	[**11,12,15,16**]
19	2-Methylpropanoic acid (isobutanoic acid)	<0.025	[8,9]	[**12**]
20	4-Methylpentanoic acid (isohexanoic acid)	X	[9]	
21	Methyldodecanoic acid	X	[9]	
22	Nonanedioic acid	X	[9]	
23	Nonanoic acid	0.05	[9]	[**12**]	
24	Octanoic acid	0.6; tr	[8,9,**15**]	[**11,12**]
25	Octenoic acid (unkn.str.)	X	[9]	
26	Pentadecanoic acid	X	[9]	
27	Pentadecen-1-oic acid	X	[9]	
28	Pentadecen-3-oic acid	X	[9]	
29	Pentadecenoic acid (branched)	X	[9]	
30	Pentanoic acid	<0.025	[8,9,**15**]	[**12**]
31	Phenylacetic acid	X	[9,10]	
32	Propanoic acid	<0.025; tr	[8,9]	[**11,12**]
33	Tetradecanoic acid	tr	[9]	[**11**]
34	Tetradecen-1-oic acid	X	[9]	
35	Tetradecen-2-oic acid	X	[9]	
36	Tetradecen-3-oic acid	X	[9]	
37	Tridecanoic acid	X	[9]	
38	Vinylbenzoic acid	X	[9]	
Alcohols				
39	(*E*)-2-Buten-1-ol	<0.01		[17]
40	(*E*)-2-Hexen-1-ol	tr		[**11**]
41	(*E*)-3-Hexen-1-ol	0.7		[**11**]
42	2(*E*)-3-Phenylprop-2-en-1-ol ((*E*)-cinnamyl alcohol)	tr	[9,10]	[**11**]
43	(*E*)-Penten-2-ol	<0.005		[**18**]
44	(*Z*)-3-Hexen-1-ol	0.1–1; 0.1; 7.0; 0.0091–0.0150, 0.005–0.05; 0.06–0.47; 0.228–0.327; 0.145–0.249;	[9,**10**,**13**,**14**,^19^,^20^,^21^]	[**11,12,15,16,**^17^**,^18^,^22^,^23^**]
45	(*Z*)-Octen-2-ol	0.005–0.05		[**12**]
46	1-Butanol	0.05; 0.01–0.1		[**12**,^17^]
47	1-Heptanol	0.005–0.05	[9]	[**18**]
48	1-Hexanol	0.01–0.1; 1.4; 0.0008–0.0029; 0.005–0.05; 0.1;	[9,10,**13**,^19^,^20^,^21^]	[**11,12,16**,^17^,**18**]
49	1-Nonanol	0.005–0.05		[**18**]
50	1-Octanol	0.005–0.05	[9,**10**,**13**]	[**18**]
51	1-Octen-3-ol	X	[**10,13,16**]	
52	1-Pentanol	<0.01; 0.005–0.05; tr	[9,**15**]	[**11**,^17^,**18**]
53	1-Penten-3-ol	0.01–0.1	[9]	[17]
54	1-Phenyl-1-propanol	X	[9]	
55	1-Propanol	<0.025		[**12**]
56	2-Butanol	X	[19]	
57	2-Heptanol	0.00073–0.00664	[9,**13**]	[**16**]
58	2-Methylbutan-1-ol	tr	[19]	[**11**]
59	2-Methylpropanol	tr	[**15**,^19^]	[11]
60	2-Nonanol	0.002–0.012; 0.003–0.007	[10]	[**22,23**]
61	2-Phenylethanol	0.5	[9,**10**,**15**]	[**11**]
62	3-Methyl-2-Buten-1-ol	0.1; 3.2	[9]	[**11,12**]
63	3-Methyl-3-Buten-1-ol	0.01–0.1		[17]
64	3-Methyl-3-Buten-2-ol	0.01–0.1		[17]
65	3-Methylbutan-1-ol	<0.025; 0.05–0.5	[9,19]	[**12**,^17^]
66	3-Pentanol	X	[19]	
67	4-Isopropylbenzyl alcohol (cuminol)	0.032–0.074; 0.023–0.064		[**22,23**]
68	4-Methyl-1-pentanol	X	[9]	
69	6-Methyl-5-hepten-2-ol	X	[9,20]	
70	Benzyl alcohol	<0.005; 3.5; 0.08–0.55; 0.00765–0.02754; 0.6	[9,10,**13,14**]	[**11,12,15,16,18**]
71	Ethanol	0.01–0.1; tr; 0.55	[19,24,25]	[**11,12**,^17^]
72	Methanol	<0.01	[19,24,25]	[17]
Phenols				
73	2-Methoxy-4-vinylphenol (4-vinylguaiacol)	tr		[**11**]
74	2-Methoxy-5-vinylphenol	tr		[**11**]
75	4-Vinylphenol	0.3		[**11**]
Aldehydes				
76	(*E*)-2-Hexenal	0.1–1; 0–0.0077; tr; 0.260–0.357; 0.289–0.425; 0.005–0.05	[**10,13,15**]	[**11,16,18,22,23**,^26^]
77	(*Z*)-3-Hexenal	2; 0.005–0.05	[10]	[**13,18**]
78	2-Heptanal	X	[20]	
79	2-Methylbutanal	X	[19]	
80	2-Methylpropanal	X	[19]	
81	2-Pentenal	0.01–0.1		[26]
82	3-Methyl-2-butenal	0.01–0.1		[26]
83	3-Methylbutanal	X	[19]	
84	3-Pyridinecarboxaldehyde (nicotinaldehyde)	X	[19]	
85	5-(Hydroxymethyl)-2-furaldehyde (hydroxymethylfurfural)	0.00143–0.00231		[**16**]
86	5-Methylfurfural	X	[**16**]	
87	Acetaldehyde (ethanal)	26	[19,24,25]	[26]
88	Benzaldehyde	0.2; 0.00165–0.00246	[9,10,**13**,^19^]	[**11,16**]
89	Decanal	0–0.00068	[9,**13**,^19^]	[**16**]
90	Heptanal	X	[9,10,19]	
91	Hexanal	0.1–1; 0.00487–0.0109; tr; 0.027–0.066; 0.090–0.172; 0.005–0.05	[9,**10**,**13**,**15**,^19^]	[**11,16,18,22,23**,^26^]
92	Nonanal	X	[9,**10**,^19^,^21^]	
93	Octanal	X	[8]	
94	Pentanal	X	[8]	
95	Propanal	<0.01		[**13**]
96	Propenal	<0.01		[**13**]
97	Undecanal	X	[9]	
Ketones				
98	(*Z*)-Jasmone	X	[9]	
99	1-Octen-3-one	X	[**10**]	
100	2,3-Butanedione (diacetyl)	0.05; <0.01	[**10**]	[**12**,^26^]
101	2-Butanone	X	[9,19]	
102	2-Decanone	X	[9,20]	
103	2-Heptanone	0.061–0.102; 0.063–0.108	[9,10,**13**,**16**,^21^]	
104	2-Hexanone	X	[9]	
105	2-Nonanone	0.005–0.05; 0.011–0.034; 0.020–0.036	[9,10,20,21]	[**18,22,23**]
106	2-Octanone	X	[9]	[**13**]
107	2-Pentanone	<0.01	[9]	
108	2-Tridecanone	X	[9]	
109	2-Undecanone	X	[9,**10**]	
110	3-Methyl-2-butanone	X	[19]	
111	3-Methyl-2-heptanone	X	[19]	
112	3-Pentanone	X	[19]	
Ketones				
113	4-Phenyl-2-butanone (benzylacetone)	X	[9]	
114	6-Methyl-5-hepten-2-one	3.2	[9,19]	[**11**]
115	3-Hydroxybutanone (acetoin)	0.1–1; 3.6; 0.125–0.749; 0.15; 0.005–0.05	[9,**13**,**14**,^19^]	[**11,12,16,18**,^26^]
116	Propan-2-one (acetone)	0.01–0.1	[**15**,^19^]	[26]
117	Acetophenone	0.00037–0.00192; 0.005–0.05	[**13**]	[**16,18**]
Lactones				
118	2-Hexen-4-olide (5-ethyl-5 *H*-furan-2-one)	0.005–0.05		[**18**]
119	Sotolon (sugar lactone)	X	[10]	
120	5-Ethyl-(3 *H*)-furan-2-one	X	[13]	
121	5-Ethyl-3-hydroxy-4-methyl-5 *H*-furan-2-one (maple furanone)	X	[10]	
122	γ-Butyrolactone (dihydrofuran-2(3 *H*)-one)	X	[9,20]	
123	γ-Hexalactone (4-hydroxyhexanoic acid lactone)	0.005–0.05; 0.7; 0.05	[9,10,20]	[**11,12,18**]
124	γ-Octalactone (4-hydroxyoctanoic acid lactone)	<0.005; 0.4; 0.05	[9]	[**11,12,18**]
125	δ-Decalactone	0.005–0.05; 0.01379–0.06106; 1; 0.666–0.917; 0.476–0.625	[9,10,**13**,^20^]	[**11,16,18,22,23**]
126	δ-Dodecalactone	0.2	[9]	[**11**]
127	δ-Hexalactone	0.6	[9]	[**11**]
128	δ-Octalactone	0.7	[9,10]	[**11,22,23**]
Furans				
129	2,5-Dimethyl-4-hydroxy-3(2 *H*) furanone (strawberry ketone)	0.1	[10]	[**11**]
130	2,5-Dimethyl-4-methoxy-3(2 *H*) furanone (berry furanone)	tr		[**11**]
131	2-Ethyl-4-hydroxy-5-methyl-3-(2 *H*)-furanone (homofuraneol)	X	[10]	
132	2-Ethylfuran	X	[19]	
133	2-Pentylfuran	X	[19]	
134	5-Methyl-4-hydroxy-3(2 *H*) furanone (norfuraneol)	0.1		[**11**]
135	Dihydroactinidiolide (apricot furanone)	X	[21]	
Esters				
136	(*Z*)-3-Hexenyl acetate	tr; 0.004–0.01; 0.003–0.011	[9,10,**13**,^19^,^21^]	[**11,22,23**]
137	(*Z*)-3-Hexenyl formate	tr		[**11**]
138	2-Methylbutyl acetate	X	[9]	
139	3-Hexen-1-yl-acetate (unkn str.)	<0.005	[20]	[**18**]
140	3-Methyl-2-buten-1-yl acetate	tr		[**11**]
141	3-Methyl-2-buten-1-yl formate	tr		[**11**]
142	3-Methylbutyl acetate (isoamyl acetate)	X	[9,10,20]	
143	Benzyl acetate	tr		[**11**]
144	Butyl acetate	X	[**10**]	
145	Ethyl 2-butenoate	X	[10]	
146	Ethyl 2-methylbutanoate	X	[**10**]	
147	Ethyl 2-methylpropanoate	X	[**10**]	
148	Ethyl 3-methylbutanoate	X	[**10**]	
149	Ethyl 2-phenylacetate	X	[**16**]	
150	Ethyl 5-hydroxydecanoate	0.8		[**11**]
151	Ethyl 5-hydroxyoctanoate	1.3		[**11**]
152	Ethyl acetate	tr	[8,**10**,**13**,^19^]	[**11**]
153	Ethyl benzoate	X	[10]	
154	Ethyl butanoate	X	[**10**]	
155	Ethyl hexanoate	0.005–0.013; 0.005–0.011	[9,**10**]	[**22,23**]
156	Ethyl octanoate	X	[10]	
157	Ethyl propanoate	X	[**10**]	
158	Hexyl acetate	X	[9,**13**,**15**,**16**,^20^]	
159	Hexyl fomate	X	[10]	
160	Methyl 2-hydroxybenzoate	X	[19]	
161	Methyl acetate	X	[9,19]	
162	Methyl hexanoate	X	[9,**10**]	
163	Methyl jasmonate	X	[**16**]	
164	Methyl nicotinate	X	[19]	
165	Methyl nonanoate	0–0.001; 0–0.001		[**22,23**]
166	Pentenyl acetate	X	[20]	
167	Propyl acetate	X	[10]	
Ether				
168	Methoxybenzene	X	[19]	
Hydrocarbons				
169	(*E*)-3-Methyl-1,3,5-hexatriene	X	[13]	
170	2-Methylbutane	X	[19]	
171	2-Methylnaphthalene	0.005–0.05	[9]	[**18**]
172	2-Methylpentane	X	[19]	
173	3-Methyl-1,3-pentadiene	X	[19]	
174	Acenaphtene	<0.005		[**18**]
175	Decane	X	[19]	
Hydrocarbons				
176	Dimethylbenzene (xylene)	tr	[9,21]	[**11**]
177	Dodecane	X	[19]	
178	Naphtalene	<0.005	[9]	[**18**]
179	Nonane	X	[19]	
180	Octane	X	[19]	
181	Pentadecane	X	[19]	
182	Pentane	X	[19]	
183	Tetradecane	X	[19]	
184	Tridecane	X	[19]	
185	Undecane	X	[19]	
Monoterpenes				
186	(*E*)-4,8-Dimethyl-1,3,7-nonatriene	X	[19]	
187	(*E*)-Linalool oxide (furan)	X	[9]	
188	(*E*)-Linalool oxide (pyran)	X	[9]	
189	(*E*)-β-Ocimene	X	[10,19]	
190	(*Z*)-4,8-Dimethyl-1,3,7-nonatriene	X	[19]	
191	(*Z*)-Linalool oxide (furan)	X	[9]	
192	(*Z*)-Linalool oxide (pyran)	X	[9]	
193	(*Z*)-Piperitol	X	[9]	
194	(*Z*)-Sabinol	0.2		[**11**]
195	(*Z*)-β-Ocimene	X	[19]	
196	1,8-Cineole (eucalyptol)	X	[9]	
197	3-Methyl raspberry ketone	X	[9]	
198	Terpinen-4-ol	0.05–0.5; 0–0.00644; 0.5; 0.100–0.201; 0.096–0.172	[9,**13**,**15**,^20^,^21^]	[**11,16,18,22,23**]
199	Cadinene	x	[19]	
200	Camphene		[9,19,20]	
201	Camphor	0.005–0.05		[**18**]
202	Cyclocitral	x	[9]	
203	Dihydroactinidiolide	x	[9]	
204	Dihydrolinalool	x	[9]	
205	Eugenol	tr	[**10**]	[**11**]
206	Geranial	0.005–0.05	[9,20]	[**18**]
207	Geraniol	0.1–1; 0.5; 0.05–0.5; 0.16–1.93; 0.15; 0.102–0.172; 0.121–0.167; 0.00209–0.00778	[9,10,**13**,**14**,^20^,^21^]	[**11,12,13,15,18,22,23**,^26^]
208	Isopiperitenone	X	[9]	
209	Limonene	tr; 0.001–0.002; 0.002	[**10**,**13**,^19^]	[**11,22,23**]
210	Linalool	0.005–0.05; 0.8; 0.01–0.92; 0.00124–0.01126; 0.15; 0.031–0.044; 0.008–0.015	[**10,13,14**,^17^,^20^,^21^]	[**11,12,15,16,18,22,23**]
211	Linalool oxides	tr		[**11**]
212	Linalyl acetate	X	[10]	
213	Menthene	X	[9]	
214	Menthol	tr		[**11**]
215	Menthyl acetate	X	[9,20]	
Monoterpenes				
216	Myrtenol	0.00007–0.00133	[9]	[**16**]
217	neo-Allo-ocimene	X	[10]	
218	Neral	<0.005	[9,20]	[**18**]
219	Nerol	0.005–0.05; 0.5; 0.019–0.037; 0.015–0.027	[9,20,21]	[**11,18,22,23**]
220	*p*-Cymene (1-isopropyl-4-methylbenzene)	0.9; 0.00011–0.00034; 0.008–0.023; 0.012–0.024	[9,**13**,^20^,^21^]	[**11,16,22,23**]
221	*p*-Cymene-8-ol	X	[9,20]	
222	Piperitone	0.005–0.05; 0.2	[9]	[**11,18**]
223	*p*-Menthen-2-ol	0.005–0.05		[**18**]
224	Raspberry ketone (4-(4-Hhydroxyphenyl)butan-2-one)	3.1; 1.09–4.20	[9,10,**14**]	[**11,15**]
225	Sabinene (thuj-4(10)-ene)	tr; 0.013–0.032; 0.015–0.030	[10]	[**11,22,23**]
226	Terpinolene	0.7; 0.001–0.004	[9,20]	[**11,22**]
227	Vanillin	tr	[10]	[**11**]
228	3,4-Dimethoxybenzaldehyde (methylvanillin)	tr		[**11**]
229	4-Methoxybenzaldehyde ( *p*-anisaldehyde)	X	[19]	
230	Verbenone	X	[9]	
231	Zingerone	0.3; 0.059–0.234	[10]	[**11,22**]
232	α-Cyclogeranyl acetate	X	[13]	
233	α-Phellandrene	tr; 0–0.0002; 0.026–0.100; 0.028–0.057	[9,10,**13**,^20^,^21^]	[**11,16,22,23**]
234	α-Pinene	tr; 0.011–0.027; 0.025–0.033	[9,10,**13**,**16**,^19^,^21^,^22^]	[**11,22,23**]
235	α-Terpinene	0.011–0.027; 0.004–0.025		[**22,23**]
236	α-Terpineol	0.7; 0.012–0.022; 0.035–0.058	[9,10,20]	[11,16,22,23]
237	β-Myrcene	0.001–0.008; 0.004–0.006	[9,10,**13**,^19^,^20^,^21^]	[22,23]
238	β-Phellandrene	X	[9,13,20,21]	
239	β-Pinene	0–0.0005	[9,10,13,20,21]	[**16**]
240	γ-Terpinene	0–0.018; 0–0.00016; 0.008–0.025	[9,10,**13**,^20^,^21^]	[**16,22,23**]
241	δ-3-Carene	X	[19]	
Sesquiterpenes				
242	(E)-α-Bergamotene	X	[10]	
243	(E)-β-Caryophyllene	0.15; 1.2	[9,**13**,**16**,^19^,^20^]	[**11,12**]
244	Caryophyllene oxide	0–0.00933	[**13**]	[**16**]
245	Humulene	tr	[9]	[**11**]
246	α-Caryophyllene	X	[19]	
247	α-Copaene	X	[19]	
248	α-Elemene	0.1		[**11**]
249	α-Farnesene	X	[19]	
250	α-Muurolene	X	[19]	
251	β-Bourbonene	X	[19]	
252	β-Cubenene	X	[19]	
C13-norisoprenoids				
253	(*E*)-β-ionone-5,6-epoxide	X	[13]	
254	3,4-Didehydro-β-ionone	X	[13]	
255	4-Oxo-β-ionone	X	[10]	
256	Cyclo-ionone I/edulan	X	[13]	
257	Dehydro-β-ionone	X	[13,21]	
258	Dihydro β-ionol	X	[10,13]	
259	Dihydro-α-ionone	X	[9,20]	
260	Dihydro-β-ionone	0.1; <0.005	[9,10,20]	[**12,18**]
261	Epoxy-β-ionone	0.005–0.05		[**18**]
262	Theaspirane (unkn str.)	0.005–0.05		[**18**]
263	Theaspirane A	0.00006–0.00035		[**16**]
264	Theaspirane B	0–0.00037	[**13**,^20^]	[**16**]
265	Theaspirane I	X	[9,21]	
266	Theaspirane II	X	[9,21]	
267	α-Ionol	0.00093–0.00811	[10,**13,14**]	[**16**]
268	α-Ionone	0.1–1; 0.4; 0.05–0.5; 0.72–1.81; 0.00869–0.01848; 0.95;0.023–0.052; 0.053–0.089	[9,10,**13,14**,^20^,^21^]	[**11,12,13,15,16,18,22,23**]
269	β-Damascenone	<0.005	[9,**10,13,14**]	[**18**]
270	β-Ionone	<0.01; 0.5; 0.05–0.5; 0.55–2.32; 0.00858–0.03146; 0.9; 0.056–0.093; 0.073–0.094	[9,**10,13,14**,^20^,^21^]	[**11,12,13,15,16,18,22,23**]
271	β-Ionol	0.00018–0.00595		[**16**]
Sulfur				
272	2-Methylthiophene	X	[10]	
273	Dimethyl disulfide	X	[**10**]	
274	Dimethyl sulfoxide	X	[**27**]	
275	Dimethyl sulfone	X	[**27**]	
276	Dimethyl sulfide	X	[10,24]	
277	Methional	X	[**10**]	
278	Thiophene	X	[10]	
Amine				
279	N-Methylene-ethanamine	X	[19]	

* Concentrations obtained through: direct analysis of distilled fraction from mashed fruit [26]; solvent extraction of oil from distillation of mashed fruit [12,17,18]; solvent extraction of mashed fruit [11,15,16]; SBSE of aqueous fraction from mashed fruit [22,23]. References in which identity of compound has been confirmed by comparison with authentic standard are reported in bold. ”X” refers to identified but not quantified compounds. “tr” stands for compound quantities reported as “trace amount”.

## 2. Volatile Compounds in Raspberry

Raspberry volatiles are important for the perception of sensory quality (odor, flavor) and for mold resistance [13], and some are claimed to have nutraceutical properties [28,29], although the nutritional role of volatile compounds is still very controversial. Fruit volatile compounds are influenced by numerous factors including cultivar variation, climate, soil, ripeness, and many other variables [11,19,30]. Early studies focused on isolation and identification of these volatile compounds in particular those most likely related to raspberry aroma, later moving attention to the factors affecting volatile composition and their possible biological roles.

Since terms such as aroma and flavor are extensively used in the literature reviewed, sometimes to indicate volatile compounds in general, before we begin we would like to draw attention to the significance of these terms.

The term “aroma” refers to an odor, or to a compound responsible of an odor, with a pleasant or unpleasant connotation [31]. Odor is a sensation perceived by the olfactory receptors in sniffing certain volatile substances (ortho-nasal route).

The term “flavor” refers to “a perception resulting from stimulating a combination of the taste buds, the olfactory organs, and chemesthetic receptors within the oral cavity” [32], in other words it is the combination of taste and smell features (through the retro-nasal route).

The volatile compounds released by raspberry are free forms of different metabolites, most of them often in the form of glycoside bound to sugars. The presence of glycosidically-bound volatile compounds in plants is well established [33]; they are able to release free volatile compounds by enzymatic or chemical cleavage during plant maturation, industrial pretreatments or processing and may be considered potential aroma precursors [34].

### 2.1. Free Forms

Between the 1957 and 1971 researchers at Firmenich published a series of studies describing the isolation, fractionation and identification of volatile compounds in raspberry [8,17,18,26,35,36,37,38] (see Table 1). In these studies, the product derived from juice or purée distillation of fresh fruits was extracted using an organic solvent (pentane or benzene), concentrated to obtain the raspberry oil [26,35] and further extracted with ether, then each fraction (aqueous distillate, neutral ether extract and acid ether extract) was analyzed separately. Fourteen carbonyl compounds were isolated from the aqueous distillate using 2,4-dinitrophenylhydrazine reaction and separated by paper chromatography. The compounds were identified by infrared spectroscopy: diacetyl, acetoin, acetaldehyde, 2-propenal, acetone, propanal, 3-methylbut-2-enal, 2-pentenal, (*Z*)-3-hexenal, 2-hexenal, 2-pentanone, hexanal, α-ionone and β-ionone [26]. Eleven alcohols were isolated from the ether extract of the distillate, separated by paper and column gas-chromatography, and identified by IR spectroscopy: 3-penten-1-ol, geraniol, (*Z*)-3-hexen-1-ol, hexan-1-ol, 3-methyl-3-buten-1-ol, 3-methyl-2-buten-1-ol, pentan-1-ol, butan-1-ol, (*E*)-2-buten-1-ol, ethanol, methanol [17]. The paper and column gas-chromatography (polar phase: Carbowax 20M) of the acidic fraction of raspberry oil allowed the separation of 11 acids and an ester, which were then identified by means of IR spectra: formic, acetic, hexanoic, octanoic, propanoic, butanoic, iso-butanoic, pentanoic, iso-pentanoic, 2-hexenoic, 3-hexenoic acids and ethyl acetate [8]. Finally, a further 39 compounds were identified in the neural fraction by GC-MS using columns with polar stationary phases (Chromosorb W and Carbowax 20M) and IR spectra [18] (Table 1).

Pyysalo compared the volatile compounds of a cultivated raspberry (*Rubus idaeus* cv. Ottawa) with a hybrid obtained by crossing raspberry (*Rubus idaeus*, L.) with arctic bramble (*Rubus arcticus*, L.) [12]. The volatile compounds were isolated from the press juice of the berries in a continuous vacuum evaporator, and separation, identification and quantification were than performed in three stages. The carbonyl compounds were determined as 2,4-dinitrophenylhydrazones, while the volatile acids and the neutral components were determined separately in a combined gas chromatograph-mass spectrometer using glass capillary columns coated with a polar phase (FFAP) [12]. More than 70 compounds were reported in the volatile fraction of the hybrid and 30 in the cultivated raspberry (the latter are reported in Table 1): identification was carried out from the recorded MS spectra.

Honkanen and collaborators obtained an extract containing neutral components and free fatty acids from press extracted berry juice of wild and cultivated (*Rubus idaeus* cv. Ottawa, Preussen) raspberries [11] using a pentane/ethyl ether extraction. A total of 75 volatile compounds were identified and quantified by GC-MS systems equipped with polar capillary columns coated with FFAP (Table 1). Compound identification was achieved by comparing the acquired MS spectra with those of the reference compounds.

A few years later, Guichard isolated volatile compounds in frozen raspberry (*Rubus idaeus* cv. Lloyd George) using three different extraction methods [9]: vacuum distillation, liquid-liquid extraction and a sorbent trapping method (Chromosorb 105). A total of 126 components were then identified by GC-MS (Table 1). The three methods were also qualitatively compared in terms of rapidity, fraction recovery and reproducibility [20]. Vacuum distillation allows a more efficient, preferential extraction of alcohols, including terpene alcohols. Liquid-liquid extraction is less reproducible but allows isolation of compounds of different classes and gives a better recovery of ionones. The sorbent trapping method uses a stream of N_2 _to strip the volatile compounds, which are then trapped on Chromosorb 105: this method is rapid and reproducible with a preferential recovery of monoterpenes [20]. In a later work, Guichard compared this trapping method [21] with the method used by Rapp and Knipser, originally optimized for wine aroma extraction [39], which also uses a stream of N_2_ but volatile components are trapped in a solvent (trichlorofluoromethane) [39]. Reproducibility was worse with Rapp and Knipser’s method than with sorbent trapping, especially for the terpene fraction [21].

Larsen and co-workers quantified 20 compounds (Table 1) considered important for raspberry aroma/flavor in 10 varieties (*Rubus idaeus* cv. Camenzind, Chilcotin, Glen Prosen, Glen Moy, Glen Clova, Meeker, Rutrago, Skeena, Vaten and Zenith) [15]. Volatile components were extracted by solvent extraction of mashed fruits, and separation and identification of selected compounds was achieved by GC-MS with a combination of columns coated with polar (Carbowax 20M) and non-polar (HP1) stationary phases [15].

Robertson and co-workers trapped the volatile compounds flushed by a zero-air gas flow over flowers or intact berries of *Rubus idaeus* cv. Glen Prosen in Haysep Q or Tenax TA tubes [19]. The trapped volatiles were than analyzed by automated thermal desorption-gas chromatography-mass spectrometry equipped with a medium polarity column (DB 1701): 61 chemical compounds were identified [19] (Table 1) by comparing retention time and acquired mass spectra with those reported in MS-libraries or published reports.

The major compound found in ripe fruit was ethyl acetate (12%–18%), followed by several terpenes (Table 1).

Malowicki and co-workers used the stir bar sorptive extraction (SBSE) to trap the volatile components from the juice of frozen raspberries (*Rubus idaeus* cv. Meeker, Chilliwack, Tulameen, Yellow Meeker, Willamette) and analyzed them by GC-MS [22,23]. Separation was carried out using a polar column (ZB-FFAP). They were able to identify 29 volatile compounds (confirmed with authentic standards) (Table 1) showing quantitative differences between cultivars and between different growing sites for the same cultivar [22].

Aprea and co-workers studied the volatile compounds released by the mashed fruits and juices of two raspberry cultivars (*Rubus idaeus* cv. Tulamen, Polka) by means of two rapid, solvent-free headspace methods: dynamic headspace by Proton-Transfer Reaction Mass Spectrometry (PTR-MS) and semi-static headspace solid phase micro-extraction (SPME) [24]. The GC-MS analysis of SPME trapped volatiles resulted in identification of 45 compounds (28 of which confirmed by authentic reference) while PTR-MS, a direct injection mass spectrometer [40], allowed the monitoring of hundreds of mass spectrometric signals, 29 of which were tentatively identified and 4 had established identities (Table 1). The same SPME-GC-MS procedure with separation carried out in a polar fused-silica capillary column (HP-Innowax) was used to compare the volatile profiling of different raspberry varieties (*Rubus idaeus* cv. Anne, Autumn Bliss, Caroline, Heritage, Himbo Top, Josephine, Opal, Pokusa, Polana, Polesie, 2 Polka accessions, Popiel, Tulameen) [13]: the 45 compounds identified are reported in Table 1.

Vrhovsek *et al.*, quantified 39 volatile compounds (Table 1) in a solvent extract of 5 raspberry varieties (*Rubus idaeus* cv. Autumn Treasure, Glen Ample, Himbo Top, Rubyfall and Sugana) using GC-triple quadrupole MS/MS [16]. GC separation was performed on a polar (VF-WAXms) capillary column. The five varieties investigated differed in both qualitative and quantitative volatile compound composition.

**Figure 1:**
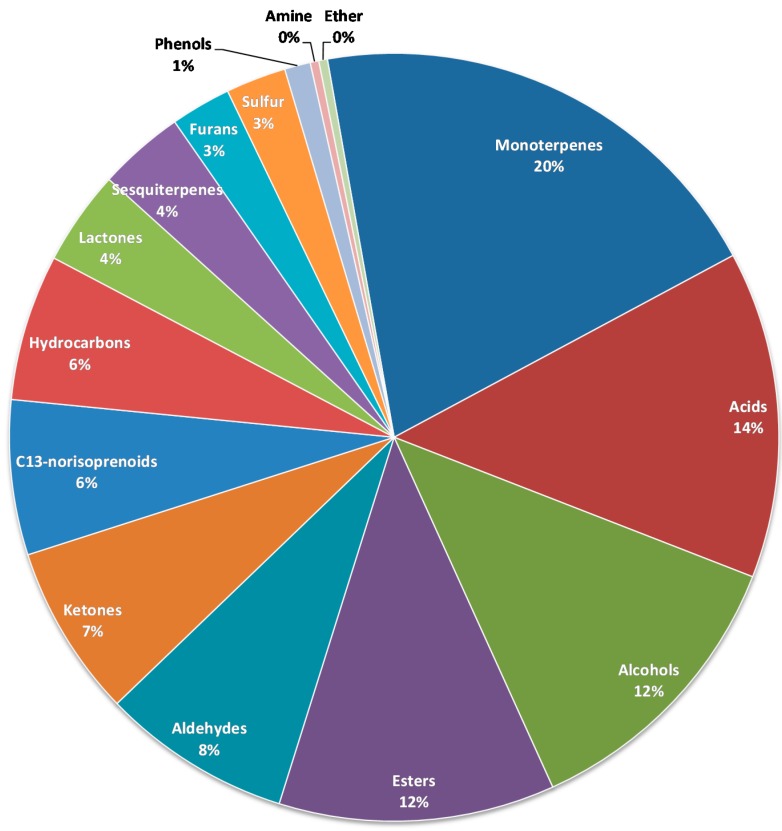
Volatile compounds reported in raspberry fruit (*Rubus idaeus* L.) according to chemical class.

A total of 276 volatile compounds are reported in Table 1 (note that three entries refer to undetermined isomers of reported compounds). Data are collected from the 20 papers dealing with raspberry fruit volatile compounds reviewed in this section. Of the 276 volatile compounds reported in raspberry, 141 have been confirmed by comparison with authentic standards (references in bold in Table 1). Figure 1 shows distribution of the 276 volatile compounds according to chemical class.

The largest class of compounds is constituted by the 56 monoterpenes reported (we use the term terpene to indicate both terpenes and terpenoids). Of these, terpinen-4-ol, geraniol, linalool, limonene, nerol, *p*-cymene, terpinolene, α- and β- phellandrene, γ-terpinene and α- and β- pinene are the most frequently reported. Terpenes derive from the common building unit isopentenyl diphosphate (IDP) and its isomer dimethylallyl diphosphate (DMADP) [41]. In plants, two parallel pathways lead to the formation of both IDP and DMADP: the mevalonate (MVA) pathway, which is active in the cytosol, and the methylerythritol 4-phosphate (MEP) pathway, which is active in the plastid. It is generally acknowledged that monoterpenes are synthesized in the plastids whereas sesquiterpenes are produced in the cytosol [42], with some exceptions [43].

A total of 38 volatile acids are reported in Table 1. A characteristic of raspberry volatile composition is the relatively high amounts of some of these volatile acids. Acetic acid is reported to be between 20 ppb and 135 ppm, the wide variability mainly attributed to variety differences [15]. Hexanoic and octanoic acids are reported at concentrations up to 19.3 ppm and 600 ppb, respectively.

Only three of the 11 sesquiterpenes found in raspberry fruits were quantified: (*E*)-β-caryophyllene, α-elemene and caryophyllene oxide.

Ten C13-norisoprenoids have been reported in raspberry up to now. These compounds are generated by oxidative cleavage of the carotenoids [44] and most of them are known to be important contributors to raspberry fruit aroma (see also paragraph 4.1).

Some compounds were reported by only one or two authors, others more frequently. Nine compounds were reported in at least half the cited works: (*Z*)-3-hexen-1-ol; 1-hexanol; hexanal; (*Z*)-3-hexenyl acetate; terpinen-4-ol; geraniol; linalool; α-ionone; β-ionone. The reported concentrations of these compounds are generally all above 100 ppb, except for (*Z*)-3-hexenyl acetate (between 3 and 11 ppb). The first four molecules, (*Z*)-3-hexen-1-ol, 1-hexanol, hexanal, (*Z*)-3-hexenyl acetate, are commonly produced by plants and are generally indicated as leaf compounds; all are characterized by green odors which have a higher intensity in (*Z*)-3-hexen-1-ol. Terpinen-4-ol, geraniol, linalool are among the major terpineols contributing to the floral scent found in fruits [45]. Other nine monoterpenes and terpenoids are reported at concentrations above 100 ppb (Table 1). α-Ionone and β-ionone are two important carotenoid-derived aroma volatile compounds [46] responsible for floral notes; indeed the odor of β-ionone is described as raspberry-violet [47].

Concentrations of volatile compounds observed by various authors in raspberry fruits can vary several-fold (Table 1). These differences are generally attributed to particular characteristics of raspberry cultivars or non-homogeneity of the fruit ripening stage. In one of the most recent works [16], the concentrations of the different volatile compounds were, in most cases, reported to be two orders of magnitude lower than previous findings. Direct comparison of the quantities reported by different authors is not feasible because of the different extraction methods and quantification procedures used. Most papers do not report recoveries of the extraction method used, and when carrying out quantitative procedures the matrix effect is most of the time not taken into account when calibration curves are built.

### 2.2. Glycosidically-Bound Compounds

A large number of volatile compounds, several of them with odor activity, are glycosylated and accumulate as non-volatile and flavorless glyco-conjugates in plant tissues [48]. These glycosidically-bound compounds are present in several fruits [48] and their occurrence is typically two to eight times greater than that of their free forms [49,50].

Pabst and collaborators studied glycosidically-bound volatiles in raspberry fruit (*Rubus idaeus* cv. Heritage) after enzymatic hydrolysis [51]. In total, 57 bound aglycons originating from fatty acid, phenylpropanoid, and terpene metabolisms were separated by GC on a Chrompack fused silica CP-Wax-58-CB WCOT capillary column and identified by MS using reference standards. Terpenes and C13-norisoprenoids were the largest classes with 14 and 12 compounds each respectively, followed by alcohols with 11 compounds, and acids with nine compounds. The other 11 compounds were seven phenols, one furane, one ketone and three lactones (Table 2).

More recently, Vrhovsek *et al.* quantified the amounts of 24 glycosidically-bound compounds in five raspberry varieties (*Rubus idaeus* cv. Autumn Treasure, Glen Ample, Himbo Top, Rubyfall and Sugana) [16]. This group developed a selective GC/MS/MS method for quantitative metabolite profiling of volatile compounds in apple, grapes and raspberries [16]. The volatile compounds were extracted from frozen powder of the fruits according to the solid phase extraction method reported in previous works [49,52]. Compound separation was carried out on a polar (VF-WAXms) capillary column. The compounds belonging to several classes, alcohols (4), aldehydes (4), terpenes (4), C13-norisoprenoids (7), one sesquiterpene, one acid, one ester, one lactone and one ketone, are reported in Table 2 with quantitative information. Several of the compounds identified are present in bound form with concentrations 2 to 40 times higher than their free forms (Table 2). In the cultivar Autumn Treasure the bound form of benzyl alcohol is 44.7 times that of the free form. The amount of α-ionol present as glycol-conjugate is 40.7 and 27.5 times that of the free form in Himbo Top and Rubyfall varieties, respectively. β-damascenone was found only in bound form in the varieties investigated.

**Table 2:** Glycosidically-bound volatiles reported in raspberry fruit (*Rubus idaeus* L.).

Compounds *^a^*	Literature	
	from [51]	from [16] (mg/Kg) *^b^*
Acids		
(*E*)-Cinnamic acid	X	
(*Z*)-Cinnamic acid	X	
3-Methylbutanoic acid	X	
Acetic acid	X	
Benzoic acid	X	
Butanoic acid	X	
Hexadecanoic acid	X	
Hexanoic acid	X	0.01775–0.42944
Octanoic acid	X	
Terpenes and Sesquiterpenes		
(6*E*)-8- Hydroxylinalool	X	
Homovanillyl alcohol	X	
4-Terpineol	X	
Eugenol	X	
Geraniol	X	0.01254–0.03740
Isoeugenol	X	
Linalool	X	0.00189–0.02361
Myrtenol	X	0.00021–0.00225
Nerol	X	
Propiovanillone	X	
Raspberry ketone	X	
Vanillin	X	
Zingerone	X	
α-Terpineol	X	
*p*-Cymene		0–0.00003
Caryophyllene oxide		0.00103–0.02543
*Alcohols*		
(*E*)-2-Hexen-1-ol	X	
(*E*)-Cinnamyl alcohol	X	
(*Z*)-3-Hexen-1-ol	X	0.00197–0.00692
1-Hexanol	X	0.00291–0.01024
1-Octanol	X	
1-Phenylprop-2-en-1-ol	X	
2-Heptanol	X	0.00144–0.00561
2-Phenylethanol	X	
3-Phenyl-1-propanol	X	
6-Methyl-5-hepten-2-ol	X	
Benzyl alcohol	X	0.09488–0.36197
Phenols		
Phenol	X	
2-Methoxy-4-vinylphenol	X	
Dihydroconiferyl alcohol	X	
4-Methylphenol	X	
4-Vinyl-2,6-dimethoxyphenol	X	
4-Vinylsyringol	X	
Tyrosol	X	
Aldehydes		
Benzaldehyde		0.00195–0.01468
Decanal		0.00072–0.00173
Hexanal		0.00169–0.00557
5-(Hydroxymethyl)furfural		0–0.00110
C13-norisoprenoids		
3,4-Didehydro-β-ionone	**X**	
3-Hydroxy-5,6-epoxy-β-ionone	X	
3-Hydroxy-α-ionone	**X**	
3-Hydroxy-β-damascone	**X**	
3-Hydroxy-β-ionone	**X**	
3-Oxo-7,8-dihydro-α-ionol	**X**	
3-Oxo-α-ionol	**X**	
4-Hydroxy-β-ionone	**X**	
4-Oxo-β-ionol	**X**	
Theaspirane A	**X**	0.00024–0.00188
Theaspirane B	**X**	0.00024–0.00185
α-Ionol	**X**	0.00506–0.19229
β-Damascenone		0–0.00062
β-Ionol		0.00013–0.00667
β-Ionone		0.00037–0.00187
α-Ionone		0.00018–0.00063
furane, ketone, esters and lactones		
2,5-Dimethyl-4-hydroxy-3(2*H*) furanone	**X**	
4-Hydroxyacetophenone	**X**	
δ-Decalactone	**X**	0.00554–0.02189
δ-Octalactone	**X**	
(*Z*)-Jasmone		0.00025–0.00127
Methyl 2-hydroxybenzoate		0.00007–0.00023

*^a^* The identity of compounds has been confirmed by comparison with authentic standards but for 3-hydroxy-5,6-epoxi-β-ionone. *^b^* Quantitative data obtained after solvent extraction of mashed fruit by GC-triple quadrupole methods using authentic standards. ”X” refers to identified but not quantified compounds.

### 2.3. Enantiomeric Distribution

Natural volatile molecules are generally found with one enantiomer predominating, attributable to stereoselectively controlled biogenetic formation mechanisms [53]. It is also known that certain enantiomeric chemicals have different sensory properties in terms of both odor quality and intensity [54]. Therefore, knowing the enantiomeric distribution of chiral compounds may help in understanding aroma perception. Furthermore, enantioselectivity and isotope discrimination during biosynthesis have been recognized as important indicators of authenticity of the natural product [55] and as such represent a useful method for differentiating natural raspberry products from those adulterated with synthetic aromas [56].

Nitz and co-workers determined the enantiomeric distribution of seven γ-lactones in deep-frozen raspberry (unknown variety) by multi-dimensional GC with achiral-chiral column combinations [57]. They found γ-octalactone and γ-hexalactone predominating with 65 and 30 ppb, respectively, and a prevalence of (*S*) enantiomers. The other γ-lactones had concentrations of 5 ppb or less with a prevalence of (*S*) enantiomers for hepta-, nona- and undeca- lactones, while a racemic distribution was observed for the deca-, and dodeca- lactones.

Werkhoff and co-workers found that 99.9% of α-ionone is present in raspberry as (*R*) enantiomer [58]. The same result was obtained by Casabianca and Graff, who studied the enantiomeric distribution of α-ionone and δ-decalactone in three raspberry cultivars (*Rubus idaeus* cv. Mecker, Heritage and Williamette) and commercial raspberry products (tea, syrup and juice) [56]. One enantiomer was predominant in raspberry fruit while commercial product prepared with synthetic flavors displayed a racemic distribution of the two enantiomers. The (*R*) enantiomer of α-ionone was found to be more than 98%. In contrast, the (*S*) form was more than 98% in δ-decalactone. The results for α-ionone were corroborated by Sewenig *et al.* [59] in different raspberry varieties (*Rubus idaeus* cv. Rucami, Schönemann-Meyer, Meeker, Rumiloba, Glen Ample and Tulameen).

Malowicki and co-workers reported the isomeric ratios of α-ionone, α-pinene, linalool, terpinen-4-ol, δ-octalactone, δ-decalactone and 6-methyl-5-hepten-2-ol in several raspberry varieties (*Rubus idaeus* cv. Meeker, Chilliwack, Tulameen, Yellow Meeker, Willamette) [22]. Isomeric ratios for lactones and α-ionone were in agreement with previous studies. Linalool was almost a racemic mixture, with a slightly higher percentage of the (*S*)-isomer. In terpinen-4-ol the (*S*) isomer was about 80%. For 6-methyl-5-hepten-2-ol it was not possible to ascertain which enantiomers eluted first and were indicated as enantiomer 1 with abundances from 77% to 86% and enantiomer 2. Finally, α-pinene was found to be present only as (*R*)-isomer. Table 3 summarizes the enantiomeric distribution of the selected compounds isolated from raspberry fruits reviewed in this section.

**Table 3:** Enantiomeric composition of selected compounds reported in raspberries (*Rubus idaeus* L.).

Compound	Enantiomer		Reference
	*R* (%)	*S* (%)	
γ-Hexalactone	34	66	[53]
γ-Heptalactone	25	75	[53]
γ-Octalactone	44	56	[53]
γ-Nonalactone	28	72	[53]
γ-Decalactone	49	51	[53]
γ-Undecalactone	55	45	[53]
γ-Dodecalactone	50	50	[53]
δ-Octalactone	0–6	94–100	[32]
δ-Decalactone	0–2	98–100	[52]
	0–3	97–100	[32]
α-Ionone	99.9	0.1	[54]
	98–100	0–2	[52]
	>99.9	-	[55]
	97–99	1–3	[32]
α-Pinene	100	0	[32]
Linalool	36–51	49–64	[32]
Terpinen-4-ol	18–21	79–82	[32]

### 2.4. Formation and Development

#### 2.4.1. Development during Ripening

Fruit ripening is a highly coordinated, genetically programmed, irreversible phenomenon involving a series of physiological, biochemical, and sensory changes that lead to the development of a soft, edible ripe fruit with desirable attributes [60]. During ripening the odor and flavor of the fruits develop through the production of several volatile and non-volatile compounds (sugars, acids) and/or degradation of bitter principles (flavonoids, tannins, and related compounds) [61].

Guichard followed the evolution of different volatile compounds in two raspberry varieties (*Rubus idaeus* cv. Lloyd George and Rose de Côte d’Or) during ripening [62]. Four stages of ripening were identified: green-pink, pink, ripe and over-ripe. In both varieties all the terpenes and sesquiterpenes measured (α-pinene, β-pinene, myrcene, α-phellandrene, p-cimene, β-phellandrene, γ-terpinene, caryophyllene and humulene) greatly increased during ripening. The chromatographic peak areas varied from 10 to 1000 for the different terpenes and sesquiterpenes. Esters (isopentyl-, pentenyl-, (*Z*)-3-hexenyl- and methyl- acetate) also increased 10–100 fold during ripening. Geraniol was at its highest at the ripening stage in the cultivar Lloyd George but continued to increase up to the over-ripe stage in the cultivar Rose de Côte d’Or. Dihydro-β-ionone was at its highest at the ripe stage then decreased. *α*-Ionone increased slightly during ripening in both varieties, while β-ionone increased slightly only in Lloyd George and not at all in Rose de Côte d’Or.

Robertson and co-workers included the flowering stages in their study of the evolution of volatile compounds in raspberry [19]. They sampled volatile compounds at six sequential stages of inflorescence development in the raspberry (*Rubus idaeus*) cultivar Glen Prosen: green buds, flowers, old flowers/early green fruit, green fruit, pink fruit, mature red fruit. During raspberry ripening, the saturated aldehydes from six to 10 atom carbons increase steadily, as did several monoterpenes such as α-pinene, camphene, α-phellandrene and limonene, in agreement with previous observations [62]. However, the two terpenes (*E*)- and (*Z*)-ocimene and the ester (*Z*)-3-hexenyl acetate greatly decreased. The terpene α-copaene and the sesquiterpene β-caryophyllene reached a maximum at the green stage then decreased considerably during berry ripening. The two ionones α- and β- only appeared during the last two stages of ripening as did the three esters methyl acetate, propyl acetate and ethyl hexanoate [19].

#### 2.4.2. Postharvest Development

Boschetti *et al.* measured the volatile compounds in raspberry [25] by direct injection method. They used a Proton Transfer Reaction Mass Spectrometer (PTR-MS) to monitor the emission of volatile organic compounds during postharvest aging of six different kinds of berry including the raspberry cultivar Tulameen. Using PTR-MS it was possible to monitor VOC emission from individual or small quantities of intact berries in real time and at high sensitivity without the need for any treatment or accumulation method [25].

Raspberries were monitored for three consecutive days before they started to decay at the end of the third day. The highest emissions recorded on the first day were methanol, acetaldehyde (4–5 ppm) and ethanol (1 ppm). Methanol, a major volatile associated with aging, reached a concentration of 40 ppm. Masses related to esters were constant and below 10 ppb over the three days [25]. It was suggested that simultaneous monitoring of the emissions of a large number of volatiles in real time and at high sensitivity can be used to describe fruit products and processing [25].

#### 2.4.3. Fast Processes

**Figure 2:**
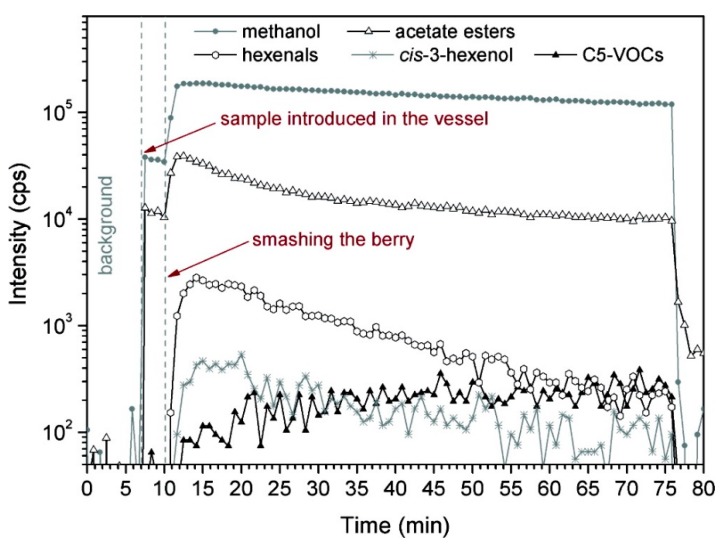
Real-time PTR-MS monitoring of VOCs released during crushing of raspberry fruit. Reproduced with permission from [24]. Copyright © 2009 American Chemical Society.

Aprea *et al.* monitored real time release of VOCs during mashing of the fruit [24] to simulate what happens during chewing or what could be the consequence of fruit damage during handling. In general, volatile emission increases after crushing of the fruits as the physical barriers trapping these secondary plant metabolites are disrupted. As well as the preformed plant metabolites, several other compounds of neo-formation (mainly oxidation product) are released. Figure 2, taken from Aprea *et al.* [24], reports the development over time of selected compounds monitored during raspberry crushing. At 10 min from the beginning of the experiment (indicated by the arrow) a sudden increase in the volatile compounds emitted occurs. Methanol, acetate ester, and acetic acid signals increase 4- to 5-fold in less than 1 min. (*Z*)-3-Hexenol increases 13-fold, while the peak in the C6-VOC signal after 4 min represents a 150-fold increase. These latter compounds together with C5-VOC are typical wounding products emitted by leaves and fruits, which originate from the lipoxygenase and hydroperoxide lyase pathways and are responsible for the typical green notes of fruits and leaves when crushed [63].

The experiment revealed that compounds produced by plant metabolism and accumulated in fruit tissue or constantly released, such as acetate esters, and compounds that are a direct consequence of tissue damage, such as C6-VOCs, have different release patterns. Since these compounds are also produced during food consumption they could affect sensory perception of the berries. The study mentioned in this section [24] was carried out using instruments coupled with a quadrupole mass analyzer which provides only the nominal mass of the observed spectrometric peaks, and therefore several interferences cannot be excluded (a version of the instrument coupled with time of flight mass spectrometry which improves the capacity of compounds identification is currently available [64]).

### 2.5. Odor-Active Compounds

Few of the many volatile compounds reported in raspberry (Table 1 and Table 2) are recognized as important for the aroma of this fruit. One of the first compounds to be identified as having an impact on the character of raspberry is 4-(4-hydroxyphenyl)butan-2-one [38], which for this reason was named raspberry ketone. This compound is synthetized in *Rubus idaeus* by condensation of *p*-coumaroyl-CoA with malonyl-CoA and successive reduction [65]. Borejsza-Wysocki and co-workers measured the content of raspberry ketone in six raspberry cultivars (*Rubus idaeus* cv. Camby, Meeker, ORUS 576-47, ORUS 2078, Royalty and Willamette) and subjected them to organoleptic evaluation [66]. The 11 judges scored the varieties on a 0–100 scale for intensity of “raspberry” flavor and aroma. The highest flavor score (56.2) was obtained for the Willamette variety, which had the highest raspberry ketone content of the six raspberry cultivars investigated [66]. In their study, Larsen and co-workers identified raspberry ketone and α- and β-ionone as the most important aromas in the 10 different raspberry varieties investigated (*Rubus idaeus* cv. Camenzind, Chilcotin, Glen Prosen, Glen Moy, Glen Clova, Meeker, Rutrago, Skeena, Vaten and Zenith) [15]. They confirmed that pure raspberry aroma was highly dependent on raspberry ketone content, while α- and β-ionone were found to be important for the overall aroma. α-Ionone in raspberry is known to be present in the (*R*)-enantiomeric form [33,34,67], which is reported to produce a violet-like, fruity, raspberry-type, floral odor [68], while β-ionone is described as “fragrant” and “floral” [69].

Klesk and co-workers investigated odor-active compounds in red raspberry cultivar Meeker by Aroma Extract Dilution Analysis (AEDA) [10]. This technique involves the flavor extract being sequentially diluted and each dilution analyzed by GC-O by a small number of judges. The flavor dilution (FD) of an odorant corresponds to the maximum dilution at which that odorant can be perceived by at least one of the judges [70]. Although FD factors do not conclusively establish that one sample contains more of a given aroma compound than another, it gives an indication of the compounds that may contribute to the overall aroma of a product. Klesk and co-workers identified 75 odor-active volatiles (see Table 1) in the Meeker raspberry cultivar from two locations in the United States (Oregon and Washington) [10]. Compound identifications were confirmed by injection of authentic standards. The most intense compounds found in both samples included strawberry furanone, hexanal, β-ionone, (*E*)-β-ocimene, 1-octanol, β-pinene, (FD 2048), β-damascenone (FD 512), acetic acid, (*Z*)-3-hexenal, methional (FD 256), (*Z*)-3-hexenol, and linalool (FD 128). Differences between the fruit from the two locations were found for other compounds [10]. As the authors themselves recognize, defining FD is only a first step towards measuring the true odor impact of these compounds [70], which would require chemical quantification of these potent odorants and generation of their OAVs to be carried out.

### 2.6. Genetic Diversity

#### 2.6.1. Wild Raspberries

In 1980 Honkanen *et al.* carried out qualitative and quantitative comparisons of the volatile compounds in Finnish wild raspberries and in two cultivated varieties (Ottawa and Preussen) using GC-MS [11]. Volatile compounds were isolated from pentane/ethyl ether in raspberry juice extract and GC separation was performed in a polar (FFAP) capillary column. A total of 75 molecules were identified (Table 1) with the aid of authentic standards. As with the cultivated varieties, volatile acids (especially acetic and hexanoic) in the wild varieties were found to be present in high concentrations (24 ppm). The authors reported the presence in wild raspberries of two acids, 3-methyl-2-butenoic and 3-methyl-3-butenoic, which have not been found in any other cultivated raspberry. A few terpenes and sesquiterpenes have been found to be specific to wild raspberries, such as (*Z*)-sabinol, menthol, and α-elemene. The alcohol fraction in wild raspberries was reported to be about twice that of the cultivated varieties (24 and 10%–15%, respectively). The two *trans* enantiomers, 2-hexen-l-ol and 3-hexen-l-ol, not found until now in cultivated berries, were also reported to be present in wild berries. Several volatile phenolic compounds were identified in the wild berries, such as 2-methoxy-4-vinylphenol, 2-methoxy-5-vinylphenol, 3,4-dimethoxybenzaldehyde, and 4-vinylsyringol, none of which has been reported in any cultivated variety. The amount of raspberry ketone (4-(4-hydroxyphenyl)butan-2-one), one of the most important compounds impacting on raspberry flavor [38], was found to be 3 times higher in wild berries than in cultivated varieties, although the amount of α- and β-ionone was 1.5–2 times lower. With the exception of ionones, the amounts of individual volatile compounds were generally 3–4 times higher in wild raspberries than in the cultivated varieties. The higher amounts of volatile compounds and the presence of several compounds only in wild raspberry species may contribute to their distinctive aroma. The authors also suggest that increased berry size as a result of breeding programs, hybridization and/or fertilization leads to a deterioration in the aroma of the berries [11].

#### 2.6.2. Differences among Cultivars

Terpenes, terpenoids and nor-isoprenoid volatile compounds are the major compounds that have been examined for the differentiation of raspberry cultivars [15,22] as they are highly related to raspberry odor and flavor [15].

Larsen and co-workers reported relatively small variations in raspberry ketone and ionones in the 10 cultivars they compared (*Rubus idaeus* cv. Camenzind, Chilcotin, Glen Prosen, Glen Moy, Glen Clova, Meeker, Rutrago, Skeena, Vaten and Zenith) [15]. Greater differences between the varieties were observed in the concentrations of linalool, geraniol, benzyl alcohol, acetoin, acetic acid, and hexanoic acid. The high variations in the three latter compounds were ascribed to differing enzymatic activity influenced by both variety and different degrees of ripeness [15].

Malowicki *et al.* reported large variations in α-ionone, β-ionone, geraniol, linalool, and (*Z*)-3-hexenol in different raspberry cultivars (*Rubus idaeus* cv. Meeker, Chilliwack, Tulameen, Yellow Meeker, Willamette) [22].

In a more recent work, Aprea *et al.* compared the head space SPME GC-MS profile of 14 different raspberry cultivars (*Rubus idaeus* cv. Anne, Autumn Bliss, Caroline, Heritage, Himbo Top, Josephine, Opal, Pokusa, Polana, Polesie, two Polka accessions, Popiel, Tulameen) over two consecutive production seasons (2006 and 2007) [13]. Volatile compounds were separated in a polar fused-silica capillary column (HP-Innowax). All fruits were harvested in the same experimental field using the same agronomic practices. Crop season strongly influenced the total volatile emissions. In 2007 the raspberries had higher amounts of volatile compounds (two fold for many varieties), which was attributed to the colder temperatures (and higher thermic excursions) recorded over the 2007 season in the experimental fields, located in Vigolo Vattaro (Trento, Italy) [13]. Similar effects due to temperature excursions were reported in previous works [30,71]. Nonetheless, the assembled data set allowed raspberry varieties to be clustered in groups of similar volatile patterns. In general, Polka and Popiel were characterized by low amounts of volatile compounds, while Caroline, Heritage, Himbo-top, and Josephine were much richer. Levels of terpene alcohols and C13-norisoprenoid compounds were found to be higher in Anne, Polana, Polesie, Polka-I, Polka-P, and Popiel, while monoterpenes and sesquiterpenes were higher in Autumn Bliss, Caroline, Heritage, Himbo Top, Josephine, Opal, Pokusa, and Tulameen. Tulameen was further differentiated for the amounts of C6 compounds (aldehydes and alcohols) and their esters [13]. In a subsequent study, advanced chemometric methods were used to classify the same 14 cultivars using both GC data and PTR-MS measurements [72]. Specifically, random forest (RF), penalized discriminant analysis (PDA), discriminant partial least Squares (dPLS) and support vector machine (SVM) were used for cultivar classification, and random forest-recursive feature elimination (RF-RFE) was used for feature selection [72]. These analyses revealed 2-heptanone, 2-heptanol, (*E*)-caryophyllene, and dehydro-β-ionone to be the most useful compounds for raspberry cultivar classification. Thus, not only terpenes and derivative compounds, as suggested in previous works [15,22], but also other classes of compounds may contribute to the characterization of raspberry cultivars. These cultivar differences are then reflected in the diverse aroma and possible defense mechanisms (see Section 2.8) of the selected raspberry varieties.

### 2.7. Environmental and Seasonal Effects

The raspberry fruit produces an array of volatile compounds with significant variations in their contents influenced by numerous factors including genotype, climate, soil, ripeness, and many other variables [2,10,11,13,19,22,30] that impact on odor and flavor.

Paterson and co-workers studied environmental and seasonal impacts over two seasons on the contents of twelve raspberry character volatiles (α-ionone, α-ionol, β-ionone, β-damascenone, linalool, geraniol, benzyl alcohol, (*Z*)-3-hexenol, acetoin, acetic, hexanoic acids and raspberry ketone) obtained from plants from the Glen Moy x Latham mapping population growing in open field or under cover (polytunnels) [14]. As reported in a previous work [13], significant seasonal variation (*p* < 0.001) was observed between field fruit for all volatiles except β-damascenone and acetoin. Seven volatiles were more abundant in polytunnel berries but β-damascenone and β-ionone were less abundant. Thus both season (2006/2007) and environment (field/polytunnel) significantly influenced the content of the monitored volatiles in raspberry fruit [14].

### 2.8. Mold Resistance

Raspberries are delicate fruits that soften and deteriorate rapidly after harvest. They are also highly susceptible to fungal diseases, particularly gray mold caused by *Botrytis cinerea* especially during postharvest storage [73]. Plants possess a range of preformed or inducible defense mechanisms, many of them involving secondary metabolites [74]. In fact, several volatile compounds are recognized for their inhibitory activity against pathogens, in particular *B. cinerea* in the case of raspberry [75,76]. Other studies have demonstrated that the same volatile compounds can have an opposite effect on pathogen development. For example, (*E*)-2-hexenal stimulates both *B. cinerea* spore germination and mycelial growth when present at low concentrations [77]. It was recently demonstrated that key strawberry aroma compounds stimulate *B. cinerea* conidial germination and some typical wound volatiles stimulate pathogen conidial germination or mycelial growth [78]. Thus, along with other resistance factors and defense mechanisms [79,80], volatile compounds seem to play a central role in mediating plant/pathogen interactions. Aprea *et al.* compared susceptibility to *B. cinerea* and volatile profiles in 14 raspberry cultivars [13] and found nine compounds to be negatively correlated with raspberry *B. cinerea* susceptibility: α-pinene, β-phellandrene, p-cymene, 2-heptanol, 4-terpineol, (*E*)-β-caryophyllene, β-damascenone, dehydro-β-ionone, and caryophyllene oxide. The authors suggested that quantification of these compounds in raspberry could be used as an indicator of fruit resistance to *B. cinerea* [13]. A subsequent study confirmed the importance of dehydro-β-ionone, 4-terpineol, *p*-cymene, (*E*)-β-caryophyllene for predicting raspberry susceptibility to *B. cinerea* [72].

## 3. Conclusions and Perspectives

Little literature on raspberry volatile compounds has appeared in the last 10 years and most of what there is concerning isolation and identification was concentrated during the period of Firmenich’s pioneering work. Later, the availability of more powerful analytical techniques allowed raspberry volatile composition to be studied in greater detail but only a few investigations looked at their roles in sensory perception and the ecological and physiological implications.

Aside from their nutraceutical properties, one of most important traits of raspberries in terms of human consumption is their pleasant aroma. Only a small fraction of the volatile compounds identified in raspberry fruits contribute to the aroma, so that distinguishing odor-active compounds from other volatile compounds is an important step in aroma research. The association between volatile compounds and aroma/flavor perception in a complex matrix, such as fruit, is not straightforward. For example, multiple volatiles are responsible for aroma/flavor sensations, combinations of volatiles yield flavors differing from those expected of individual compounds, and perception of volatiles differs in different matrices [81]. Moreover, the final sensory evaluation can even be influenced by psychological and multisensory factors. The only “instrument” which can discriminate between odor active compounds and other volatile compounds is the human nose. Therefore, the primary measure of the sensory attributes of flavor and aroma is descriptive sensory analysis, typically with trained sensory panels [81]. For all the above-mentioned reasons we think that the relationship between volatile compounds and odor and flavor in raspberry is worth further investigation using appropriate methodologies. Furthermore, there is little literature on raspberry characterization by sensory descriptive methods. In our opinion this issue should be better addressed in future research and breeding programs. For example, it would be desirable to include sensory traits and volatile compounds in research on quantitative trait loci, as has been done for apple [82,83,84].

Other aspects of raspberry research, only partially addressed in the literature and deserving more attention, relate to plant communication and plant-pathogen interaction mechanisms mediated by endogenous volatile compounds, as studied in other fruit (e.g., strawberry [78]). These studies will contribute to a better understanding of some of the natural defense mechanisms activated by plants with the aim of helping agronomists to manipulate and manage them in order to reduce the use of pesticides.

The identity, biochemical pathways and release of volatile compounds in raspberry has been widely investigated, but more studies are needed to better understand the various biological roles played by the different volatile compounds in raspberry.
